# Bilateral strangulated femoral hernia in a male: a rare surgical case report

**DOI:** 10.1093/jscr/rjae718

**Published:** 2024-11-17

**Authors:** Granit Ismaili, Michael Vaughan, Tarig Ahmed Abdelhafiz

**Affiliations:** Department of General Surgery, Sligo General Hospital, The Mall, Rathquarter, Sligo F91 H684, Republic of Ireland; Department of General Surgery, Sligo General Hospital, The Mall, Rathquarter, Sligo F91 H684, Republic of Ireland; Department of General Surgery, Sligo General Hospital, The Mall, Rathquarter, Sligo F91 H684, Republic of Ireland

**Keywords:** bilateral femoral hernia, strangulation, modification

## Abstract

Femoral hernias are at a high risk of strangulation due to their narrow necks. They are an exceptionally rare occurrence in males. In many cases, differentiation between a femoral and inguinal hernia is difficult. We present the case of a bilateral strangulated femoral hernia in a 70-year-old male. To our knowledge, there has been only one previously published report of such a case in males. Our patient presented with generalized abdominal pain and bilateral irreducible groin swellings, originally thought to be bilateral strangulated inguinal hernia. Upon initial inguinal incision, a diagnosis of a strangulated femoral hernia was made. A modified lower midline laparotomy incision was made to gain access to and diagnose both femoral hernias and allow for bowel resection and abdominal washout. Our case highlights the importance of modifying the surgical approach when encountering with a different diagnosis intraoperatively.

## Introduction

A femoral hernia is the protrusion of abdominopelvic contents through the femoral ring into the femoral canal and exiting via the saphenous opening. Its common presentation is a characteristic bulge below the inguinal ligament [1, 2]. Diagnosing a femoral hernia on physical examination is often difficult in obese patients and due to the tendency of the femoral hernia to move upward to a position above the inguinal ligament [3]. Femoral hernias are rare and only account for about 3% of all groin hernias [2]. Despite this, they are associated with significant morbidity and mortality due to risk of strangulation [4]. Although femoral hernias have a female to male predominance of 8:1, inguinal hernias are still the most common type of hernia in both genders [2]. As a result, the number of cases in the literature concerning incarcerated femoral hernias in males is very low [5].

Surgical intervention remains the only cure with a multitude of open, laparoscopic, and robotic techniques [3]. Regardless of the technique, patients should be operated upon electively as soon as possible or in an emergency setting if incarceration or strangulation.

## Case report

**Figure 1:**
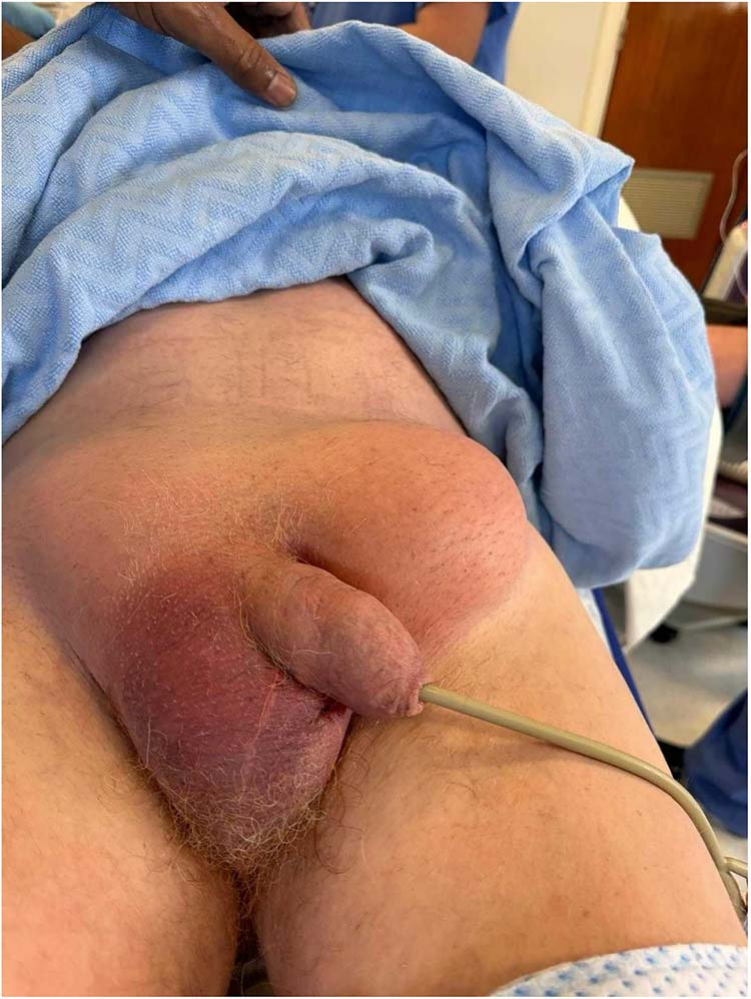
Bilateral groin swellings, larger on the left.

A 70-year-old man presented to the emergency department complaining of a 4-day history of generalized abdominal pain, bilateral groin swellings, and vomiting. He was found to be tachycardic at 130 and hypotensive at 90/50. Physical examination of the abdomen showed generalized tenderness and guarding on palpation. He had bilateral nonreducible groin swellings, the left measuring 10 × 10 cm, and the right measuring 4 × 5 cm (Fig. 1). Renal function revealed an elevated creatinine of 246 and urea of 13.4. Venous blood gas was significant for a lactate of 9. At the time, a differential diagnosis of bilateral strangulated inguinal hernias with an acute kidney injury was made.

**Figure 2:**
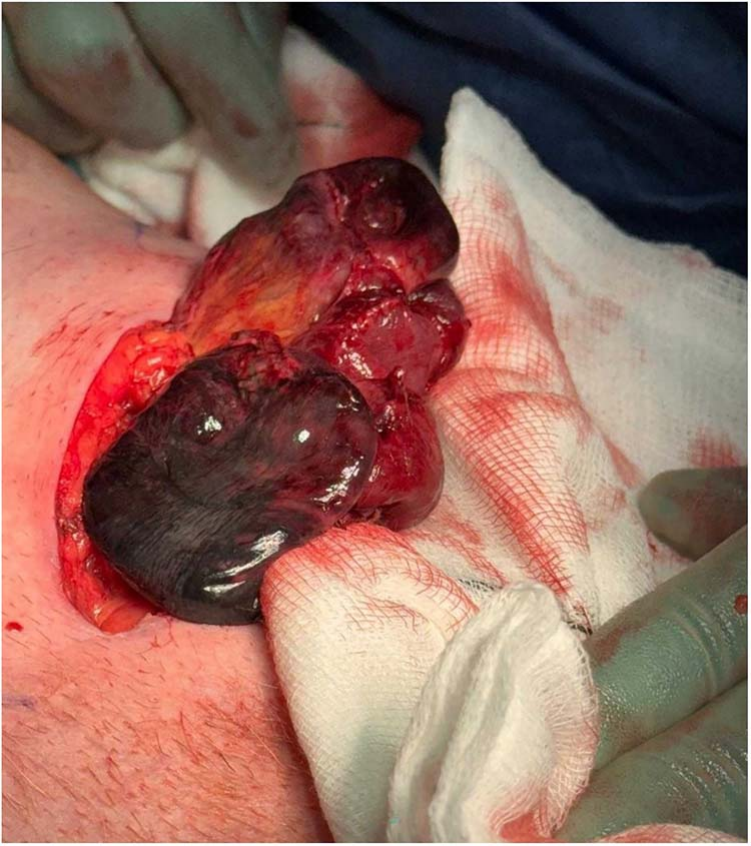
Left femoral hernia containing ischaemic small bowel.

**Figure 3:**
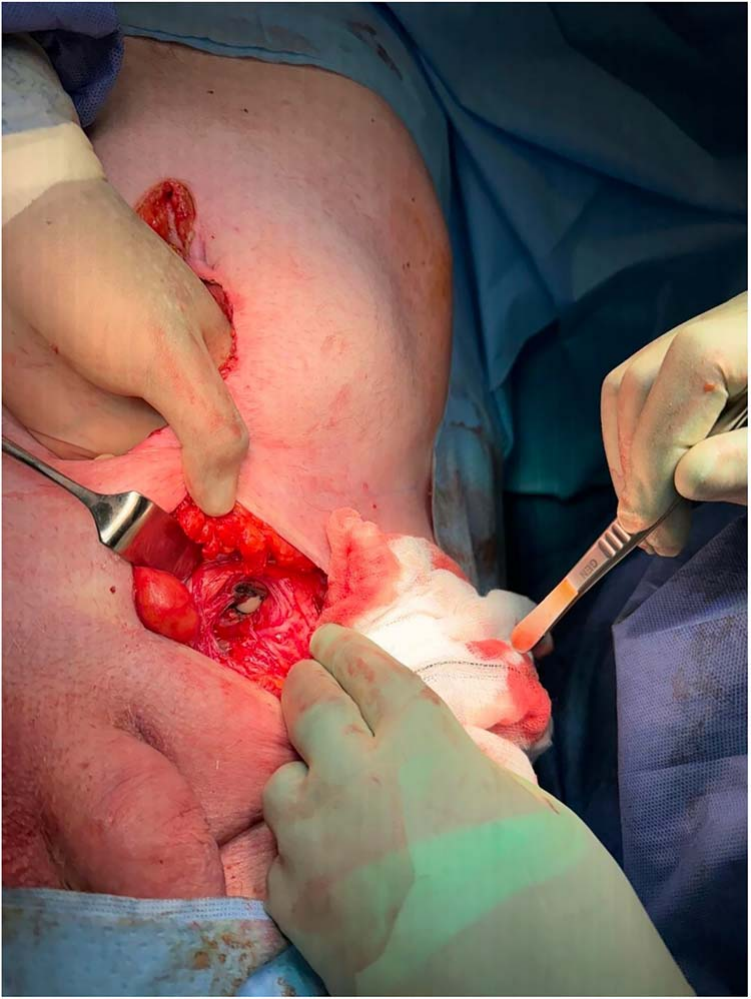
Infra umbilical midline laparotomy incision.

The patient was made nil by mouth and started on intravenous fluids, as well as intravenous piperacillin/tazobactam. Nasogastric decompression was commenced and the patient proceeded for definitive surgical management. A left inguinal incision was made, and once visualized, the hernia was noted to be below the inguinal ligament with a large section of ischaemic small bowel, in keeping with a femoral hernia (Fig. 2). An infraumbilical midline laparotomy incision was made to allow access to the bowel (Fig. 3). Upon opening, the right hernia was also noted to be a femoral hernia containing ischaemic small bowel also. The two sections of small bowel, the left hernia containing proximal ileum and the right containing distal ileum, were resected and two side-to-side anastomoses were performed. About 16 l of normal saline washout was used for the gross intra-abdominal contamination. The left femoral defect was closed with O prolene suture via the inguinal incision. Due to the increasing instability of the patient, the right femoral defect was closed with O PDS via the laparotomy incision and planned for definitive open closure electively. Two 18FR Robinson drains were placed inside the abdomen. The patient was admitted to intensive care postoperatively until full recovery and discharged to the ward after 4 weeks. He made a full recovery and was discharged home 5 weeks post initial presentation.

## Discussion

Femoral hernias are rare in males and account for 2% of all hernias in men [6]. Bilateral femoral hernias have been only reported in 10% of cases [7]. Only two cases have been reported on bilateral strangulated femoral hernias in women [8]. To the best of our knowledge, there has been only one other case in the literature of a bilateral strangulated femoral hernia in a male [9]. Physical examination can therefore present a diagnostic challenge. A differential diagnosis of a bilateral strangulated inguinal hernia was made in this case due to our clinical judgement of knowing the infrequency of femoral hernias in males. Imaging techniques such as ultrasound (US), computed tomography (CT), and magnetic resonance imaging (MRI) can be useful in diagnosis [10]. Both CT and MRI were used by Zarokosta *et al.* [9] to diagnose their bilateral femoral hernias. In our case, no radiological aid was used in order to prevent delay in surgical intervention as the patient was in critical condition.

The narrow femoral canal and rigid femoral ring give rise to femoral hernias having the highest rate of strangulation among hernias with around 15% to 20% [11]. If there is suspicion of incarceration or strangulation, then emergency surgery is needed. Surgical technique involves either an open or a minimally invasive technique. The choice depends on the surgeon’s expertise and preference, patient and hernia-related characteristics, and local availability [12]. The laparoscopic approach (transabdominal preperitoneal repair or total extraperitoneal repair) is increasingly becoming the recommended option, offering exploration of both sides and all hernia defects, enabling visualization of content and bowel for vitality [13].

Classically, three approaches are described to open femoral hernia repair: Lockwood’s infra-inguinal, Lotheissen’s trans-inguinal, and McEvedy’s high approach [14]. Of the open techniques, McEvedy’s high approach is the preferred approach in the emergency setting as this approach allows better access for visualization of bowel and possible resection is needed [14]. We initially made an inguinal incision but upon realization that the left hernia was a strangulated femoral hernia, we proceeded with a lower midline laparotomy incision. This incision also facilitated the diagnosis and management of the right strangulated femoral hernia as well as made washout of the intra-abdominal contamination possible. The decision to close the right defect unconventionally and plan for definitive elective repair on that side was made in accordance with the increasing instability of the patient intraoperatively. Regardless of the technique or approach, operative intervention should not be delayed. With very limited guidance in the literature on how to approach such a case, our ability to modify the open technique was crucial in enabling prompt surgical management. Our awareness in anatomy and pathology of femoral hernias was paramount in the survival of the patient.

## Conclusion

Femoral hernias in males pose a diagnostic challenge in distinguishing them from inguinal hernias. Performing modified versions of the open approaches allows access and visualization of the abdominal cavity and facilitates reduction of the hernias and diagnosis of other visceral abnormalities.
